# Microsatellite Instability and Mismatch Repair Subclonality in Human Cancers: Biologic Basis, Diagnostic Pitfalls, and Therapeutic Implications with a Focus on Colorectal Cancer

**DOI:** 10.3390/cancers18182955

**Published:** 2026-09-13

**Authors:** Alena A. Hasenburg, Bradley G. Somer, Sebastian Stintzing, Axel Grothey

**Affiliations:** 1 Department of Hematology, Oncology and Cancer Immunology, Universitaetsmedizin Berlin, Corporate Member of Freie Universitaet Berlin and Humboldt-Universitaet zu Berlin, 10117 Berlin, Germany; annbalou.hasenburg@charite.de (A.A.H.); sebastian.stintzing@charite.de (S.S.); 2 Department of Medical Oncology, West Cancer Center and Research Institute, Germantown, TN 38138, USA; bsomer@westclinic.com

**Keywords:** microsatellite instability, circulating tumor DNA, colorectal cancer

## Abstract

Microsatellite instability-high (MSI-H) and deficient mismatch repair (dMMR) define a molecular subtype of colorectal cancer (CRC) and predict benefit from immune checkpoint inhibition. Clinically, MSI/MMR assessment may yield discordant results across assays, anatomic sites, or time points. A challenging scenario occurs when tissue analysis identifies microsatellite-stable (MSS) or mismatch repair-proficient (pMMR) CRC, whereas circulating tumor DNA (ctDNA) analysis indicates MSI-H. This review evaluates evidence for MSI/MMR heterogeneity and subclonality in CRC. We reviewed literature on spatial, temporal and subclonal MSI/MMR heterogeneity across human cancers, focusing on CRC. Explanations for tissue–plasma and tissue–tissue discordance, including assay limitations, sampling bias, lesions misattribution, biological evolution, and treatment-related selection, were assessed. Although discordance more commonly reflects assay limitations, sampling bias, or profiling of different lesions, increasing evidence supports genuine biological heterogeneity. Distinct tumor regions may show retained MMR protein expression in one area and regional loss with MSI in another. Noncanonical MMR defects, epigenetic heterogeneity, post-treatment evolution, adaptive mutator-state, and immune selection may also generate dynamic or subclonal instability. Therapeutic relevance may depend not simply on MSI detection, but on whether the unstable clone is sufficiently dominant to generate broadly shared neoantigens across the disease burden. MSI/MMR discordance requires careful interpretation and should not automatically be considered as true biological heterogeneity. Nevertheless, genuine subclonality occurs in CRC and other cancers and may affect responsiveness to immune checkpoint inhibition. Integrated tissue, plasma, spatial and longitudinal analyses may improve treatment decisions and biomarker development.

## Simple Summary

Microsatellite instability is an important feature of colorectal cancer that can help identify patients who may benefit from immunotherapy. Test results, however, are not always consistent. For example, an initial tumor sample may show no microsatellite instability, while a later blood-based tumor DNA test suggests that it is present. In many cases, such differences are caused by limitations of the tests, differences in the samples analyzed, or testing of different tumor sites. Evidently, initial tissue-based and later ctDNA-based tests need to be validated to be able to detect MSI-H phenotype. Nevertheless, growing evidence indicates that microsatellite instability may sometimes occur only in certain parts or subgroups of a tumor and may also change during treatment. These differences could influence whether immunotherapy is effective, particularly if microsatellite instability is present in only a small proportion of cancer cells. This review summarizes the evidence for such tumor heterogeneity, explains possible causes of conflicting test results, and discusses how these findings may improve treatment decisions and future biomarker testing.

## 1. Introduction

Defective DNA mismatch repair is one of the canonical molecular routes of colorectal tumorigenesis. In CRC, loss of mismatch repair function results in accumulation of insertion and deletion mutations at microsatellite loci, a hypermutator phenotype, characteristic clinicopathologic features, and marked immunogenicity. MSI-H/dMMR CRCs account for a clinically meaningful fraction of localized disease and a smaller but highly consequential subset of metastatic disease [1,2,3,4]. The recognition of this phenotype has transformed hereditary cancer risk assessment, particularly after the demonstration that dMMR predicts sensitivity to PD-1 blockade across tumor types [5,6]. Additionally, it has transformed systemic therapy, after the subsequent establishment of pembrolizumab and nivolumab-based strategies in metastatic MSI-H/dMMR CRC [7,8,9,10].

Historically, MSI and MMR status have been treated as essentially binary variables: tumors are classified as either MSS/pMMR or MSI-H/dMMR. That framework remains clinically useful and is supported by high concordance rates across well-performed assays. Real-world practice, however, increasingly reveals cases that do not fit a binary model. These include abrupt subclonal loss of MMR protein staining by immunohistochemistry (IHC), discordant MSI and MMR calls across different tumor blocks, discordance between primary and metastatic lesions, and temporal discrepancies in which ctDNA suggests MSI-H after earlier tissue testing showed MSS/pMMR. Such findings have renewed interest in the concept of MSI/MMR subclonality: the presence, within a single patient’s cancer, of spatially or temporally restricted compartments with distinct MMR function and microsatellite instability status [11,12,13].

For the practicing molecular oncologist, the key question is not merely whether subclonal MSI can occur, but whether it is biologically meaningful and therapeutically actionable. This question matters especially in CRC, where immune checkpoint inhibitors (ICIs) can produce durable and occasionally transformative responses in canonical MSI-H/dMMR disease, yet generally have little activity in conventional MSS CRC [14]. The concern is that a narrowly subclonal MSI-H state may not be sufficient to confer the broad neoantigenic landscape and inflammatory microenvironment that underlie the efficacy of PD-1 blockade in classic dMMR tumors [13,15]. Conversely, strict adherence to an oversimplified binary model may cause truly unstable tumors to be overlooked when standard tissue assays miss spatially restricted or later-emerging MMR-deficient clones [4,16].

This review examines the concept of MSI/MMR subclonality in human cancers, focusing on CRC. We distinguish true biologic heterogeneity from analytic discordance, summarize the pathology and genomics of heterogeneous MSI/MMR states, discuss mechanisms that may generate them, review the performance and limitations of plasma MSI testing, and consider whether such cancers are likely to respond to immunotherapy. This article was conducted as a narrative literature review. No formal systemic search strategy or predefined search protocol was applied. Relevant clinical studies and review articles addressing MSI, MMR, and MSI/MMR heterogeneity across human cancers were identified and selected on the basis of their relevance to the objectives of this review. Particular emphasis was placed on evidence from CRC, while studies from other tumor types were included when they provided relevant biological, pathological, or diagnostic insights into heterogeneous or subclonal MSI/MMS phenotypes. The literature was reviewed with a focus on spatial, temporal MSI/MMR heterogeneity, regional or subclonal loss of MMR protein expression, discordance between immunohistochemistry and molecular MSI testing, tissue–tissue and tissue–plasma discordance, and the detection of MSI using ctDNA. Potential mechanisms underlying heterogeneous MSI/MMR phenotypes were evaluated, including canonical and noncanonical MMR defects, epigenetic heterogeneity, treatment-related tumor evolution, adaptive changes in mutations, and immune-mediated selection. Clinical studies investigating the relationship between MSI/MMR status, tumor heterogeneity, neoantigen clonality, and response to immune checkpoint inhibition were also considered. ChatGPT v5.5 was used to perform a comprehensive literature search and for the development of the figures.

## 2. Definitions and Conceptual Framework

MSI refers to the gain or loss of repeat units at microsatellite loci resulting from defective post-replicative DNA repair. dMMR refers to loss of function of the mismatch repair pathway, classically involving MLH1, PMS2, MSH2, and MSH6 [4]. Although the two states are tightly linked, they are not perfectly interchangeable. Some tumors with abnormal IHC do not demonstrate a canonical MSI pattern on molecular testing, and some tumors classified as pMMR by IHC are molecularly MSI-H because the retained protein is nonfunctional or because the affected repair defect lies outside the standard four-protein IHC panel [13,17,18]. Accordingly, the terms dMMR and MSI-H should be viewed as overlapping but nonidentical biomarker states rather than exact synonyms.

**Figure A1:**
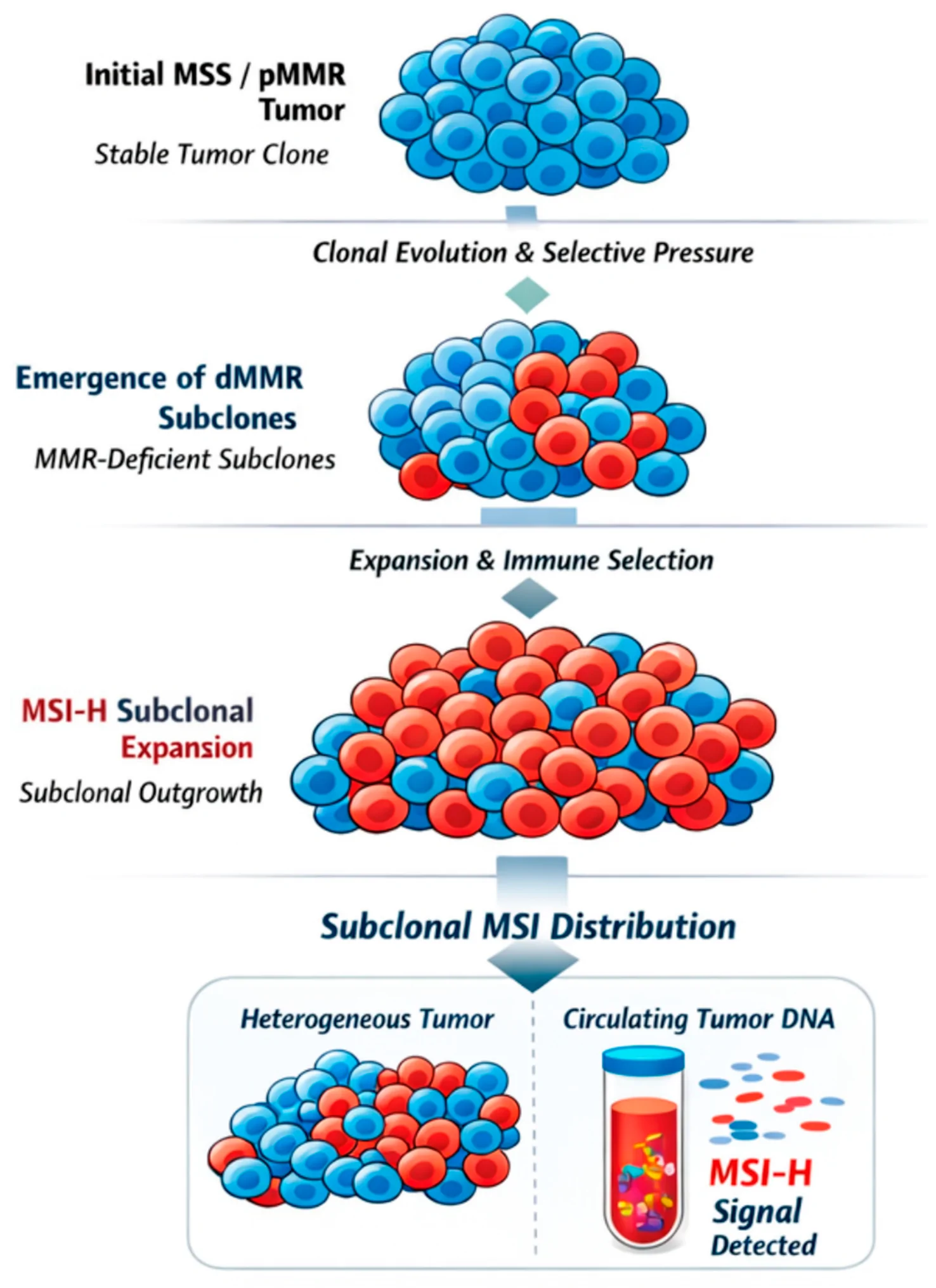
Evolutionary Model of MSI Subclonality.

The term MSI/MMR subclonality is best reserved for cancers in which distinct subpopulations of tumor cells within the same cancer differ in their mismatch repair function or microsatellite phenotype. This may be recognized pathologically as abrupt or mosaic subclonal loss of one or more MMR proteins, molecularly as region-specific MSI, or evolutionarily as emergence of an unstable branch during tumor progression (Figure A1) [4]. By contrast, discordance is a broader term that includes both true biologic heterogeneity and nonbiologic explanations such as poor fixation, low tumor content, assay-specific blind spots, specimen mix-up, interpretation error, or profiling of different primaries in the same patient. This distinction is crucial, because most clinically encountered discrepancies are not ultimately due to true subclonal biology.

A useful framework is to divide challenging cases into four categories: first, true spatial heterogeneity within a single lesion; second, temporal evolution of the mutator phenotype over the natural history of disease or under treatment pressure; third, assay phenotype mismatch, in which dMMR and MSI do not align because of the biology of the affected gene or assay limitations; and fourth, apparent discordance caused by sampling or attribution error. This framework helps interpret the clinical scenario of tissue MSS/pMMR followed by ctDNA MSI-H, which may represent any of these categories. In some cases, ctDNA may also reveal a biologically genuine MSI-H subclone that was either underrepresented in previously sampled tissue or remains a minority component of the overall disease burden.

## 3. Epidemiology of MSI Across Human Cancers and Its Relevance to Subclonality

Although this review focuses on CRC, other tumor types have helped sharpen the conceptual and diagnostic language around subclonal MSI/MMR states. In endometrial carcinoma, heterogeneous and biphasic MMR protein expression has been documented with corresponding subclonal MSI, and metastases have in some cases represented only the MMR-intact component [19].

Pan-cancer genomic studies established that MSI extends far beyond CRC and endometrial carcinoma, although prevalence varies widely by histology. In a large cross-cancer analysis, MSI was identified across dozens of tumor types, underscoring that the biology of mismatch repair deficiency is broadly relevant even if its frequency and phenotypic clarity differ across tissues. Another large exome-based study similarly showed that MSI-positive tumors can be identified and are associated with characteristic frameshift mutations across the genome. These pan-cancer observations matter for subclonality because they show that MSI is not a uniform state even within a given organ system and may be shaped by tissue context, selective pressures, and accompanying genomic programs. In CRC specifically, MSI-H tumors are enriched within the immune-rich CMS1 subtype and are commonly associated with hypermutation, *BRAF* mutation, CIMP-high status, proximal primary location, and lymphocyte-rich histology. Yet, even within CRC, consensus molecular analyses have identified mixed or indeterminate tumors whose features may reflect either transitional states or true intratumoral heterogeneity. This molecular heterogeneity in CRC provides a conceptual backdrop for understanding why MMR dysfunction may not always be clonally uniform [20]. The broader implication is that the challenge of MMR/MSI heterogeneity is not unique to CRC. Rather, CRC sits within a wider oncologic problem: MMR dysfunction may exist along a spectrum from clonal, homogeneous deficiency to focal, subclonal, or dynamically evolving deficiency.

## 4. Evidence for Intratumoral MSI/MMR Heterogeneity in CRC

The clearest early evidence that MSI/MMR heterogeneity can occur in CRC came from direct regional sampling studies. In a seminal analysis of 100 right-sided sporadic colon carcinomas, Chapusot and colleagues compared PCR-based MSI testing with IHC for MMR proteins and found initially discordant cases that resolved after analysis of additional tumor areas. Importantly, when discrepant regions were tested separately, areas showing loss of MMR protein expression were MSI-positive, whereas adjacent areas with retained expression were not. The study concluded that IHC could accurately identify unstable phenotype provided that more than one area of the tumor was evaluated, specifically because of intratumoral heterogeneity. This remains one of the foundational demonstrations that true regional MMR/MSI heterogeneity exists in CRC [12].

Subsequent pathology literature has reinforced this observation. McCarthy and colleagues described heterogeneous loss of MMR protein expression as a definable and underrecognized phenomenon that complicates both interpretation of IHC and correlation with MSI testing. Although their series included non-CRC cases, the central message is directly relevant to colorectal pathology: heterogeneous loss is a real biologic pattern, not merely a staining artifact, and careful reporting is required when abrupt, geographically distinct areas of loss are present. More recently, Riedinger and colleagues characterized MMR/MSI-discordant and subclonal cases primarily in endometrial cancer and demonstrated that heterogeneous MMR patterns arose through multiple mechanisms, including epigenetic, germline, and somatic alterations. Although these data originated in endometrial cancer, they provide an important conceptual framework for understanding analogous MSI/MMR heterogeneity in colorectal cancer. These data support the view that heterogeneous MMR loss is not a purely technical phenomenon and can arise through multiple biologic routes [15,21].

Additional support comes from modern concordance studies. In the large CRC series reported by De Salins and colleagues, discordance between MMR IHC and MSI testing was initially identified in 1.6% of 3228 cases, but after re-examination of pathology and repeat analyses, only 0.4% remained truly discordant. This finding is important for two reasons. First, it confirms that most discrepant cases are resolved by expert adjudication, implying that true heterogeneity is uncommon. Second, it shows that a small but clinically important residue of biologically complex tumors persists even after rigorous review. In effect, true subclonality is rare, but it clearly exists [16].

A particularly intriguing development has been the recognition of pMMR/MSI-H CRC as a distinct entity. Xu and colleagues, in EBioMedicine, identified a cohort of CRCs classified as pMMR by standard IHC but MSI-H by molecular analysis. These tumors displayed clinicopathologic and immune features intermediate between classic pMMR/MSS and dMMR/MSI-H CRC and showed increased CD8-positive T-cell and NK-cell infiltration relative to pMMR/MSS tumors. The study also implicated noncanonical genomic mechanisms, including an *MSH3* frameshift mutation, suggesting that at least some pMMR/MSI-H tumors are not assay artifacts but instead represent biologically unstable tumors not captured by routine four-protein IHC [22]. Such cases may overlap conceptually with what clinicians perceive as subclonal MSI, especially when tissue and liquid analyses disagree.

## 5. Mechanisms of MSI/MMR Subclonality in CRC

Regional acquisition of a second hit in a canonical MMR gene is a straightforward mechanism for subclonal dMMR. In Lynch syndrome, the founder lesion may already harbor a germline mutation, and only a subset of tumor cells may later acquire the second event needed for complete loss of repair. In sporadic CRC, analogous branching can occur through two somatic hits. Under this model, an ancestral pMMR/MSS clone gives rise to a daughter subclone that becomes dMMR and then rapidly accumulates indels and MSI. Pathology then reveals patchy or abrupt loss of protein expression, and molecular testing may vary according to which region is sampled. The mechanistic heterogeneity identified in recent characterization studies, including germline, somatic, and epigenetic causes within heterogeneous MMR cases, is consistent with this framework [4,15].

Epigenetic heterogeneity, especially involving *MLH1*, provides another route. Sporadic MSI-H CRC most commonly results from *MLH1* promoter hypermethylation. If promoter methylation is regionally acquired, incomplete, or differentially selected across tumor sectors, a lesion could contain both MLH1-silenced and MLH1-intact compartments. Such epigenetic heterogeneity would produce regional dMMR and potentially regional MSI. Recent pathology discussions from CAP explicitly note the relevance of epigenetic mechanisms in subclonal or heterogeneous MMR loss, and the molecular characterization of these cases shows that epigenetic loss is a common mechanism in heterogeneous MMR tumors [13,15].

Retained but nonfunctional MMR proteins also matter. Certain missense or C-terminal alterations, particularly in *MSH6*, may preserve antigenicity while abolishing repair function. In such cases, a tumor can appear pMMR by IHC yet behave as MSI-H by molecular testing. This mechanism does not necessarily create true subclonality, but it can mimic it when different assays or different specimens are used. It is one of the most important biologic explanations for pMMR/MSI-H CRC and for apparently contradictory tissue and plasma results [23].

Noncanonical MMR pathway defects further complicate classification. Routine CRC IHC assesses MLH1, PMS2, MSH2, and MSH6, but DNA repair extends beyond these proteins. Alterations involving *MSH3*, *PMS1*, *MLH3*, *EXO1*, and related components may perturb microsatellite stability without generating the classic four-protein IHC loss pattern. The pMMR/MSI-H CRC series by Xu and colleagues supports this concept, with evidence for alterations such as *MSH3* frameshift mutation in apparently pMMR but molecularly MSI-H CRC. These tumors are particularly relevant to the concept of subclonality because they may harbor localized or partial repair defects that produce a muted or noncanonical instability phenotype [22].

*POLE/POLD1*-driven hypermutation with secondary MMR disruption may represent a less common mechanistic bridge. Ultra-hypermutated tumors driven by *POLE* or *POLD1* exonuclease domain mutations are usually discussed separately from MSI-H cancers, but biology does not always respect such categories. CAP’s recent review highlights that polymerase proofreading defects can coexist with, or potentially precede, secondary MMR abnormalities. A tumor with an initial proofreading defect may accumulate further repair pathway damage and acquire an MSI-like phenotype in a branch-specific manner [24].

Adaptive mutator-state switching within MMR-deficient CRC is another emerging concept. Kayhanian and colleagues showed that homopolymer switches involving genes such as MSH6 and MSH3 can modulate subclonal mutation rate and diversity, effectively allowing MMR-deficient CRCs to adapt their mutability under immune selective pressure [25]. This work suggests that even within a globally dMMR tumor, subclones may differ in the intensity and pattern of ongoing mutagenesis. In practical terms, MSI is not always a static state; it may be dynamically tuned during tumor evolution.

Immune selection and the evolution of immune escape add another layer. Kobayashi and colleagues, using public and multiregion data, showed that MSI-H CRCs acquire antigen-presentation machinery alterations and HLA-related immune escape mechanisms in a subclonal fashion under Darwinian selection [26]. These findings suggest that an unstable, immunogenic clone may itself diversify under immune pressure, leading to regional differences in antigen presentation capacity and immune visibility. Thus, what appears clinically as variable immunotherapy sensitivity may partly reflect heterogeneity not only in mutational burden but also in immune escape architecture.

Finally, therapy-induced or therapy-selected evolution is biologically credible. Treatment pressure can plausibly generate or enrich a dMMR/MSI-H subclone over time. Although this remains incompletely defined in routine CRC, translational work around temozolomide-treated CRC has shown that alkylator exposure can alter the mutational landscape and intersect with mismatch repair biology. More broadly, any therapy that imposes a strong selective bottleneck could favor expansion of a pre-existing MMR-deficient branch or create conditions under which new repair defects emerge [27]. This mechanism is especially relevant in patients whose ctDNA becomes MSI-H after prolonged therapy despite earlier MSS/pMMR tissue testing.

## 6. Diagnostic Discordance: More Common than True Biologic Subclonality

Any review of MSI subclonality must emphasize that true biologic heterogeneity is much less common than apparent discordance. Current CAP/ASCO guidance recognizes IHC, PCR-based MSI testing, and NGS-based assays as complementary approaches and recommends orthogonal testing when results are unexpected or discordant. Recent CAP guidance on navigating discordant MMR/MSI results catalogues the major causes of discrepancy: low tumor content, degraded material, atypical MSH6 patterns, retained nonfunctional proteins, noncanonical repair defects, multiple primary tumors, and interpretive errors. This framework is central to clinical management because it guards against reflexively labeling all tissue–plasma disagreements as biologic conversion [13,17,28].

The Geurts study provides an important cautionary clinical perspective. In patients entered into a molecularly guided treatment program with tumors considered dMMR/MSI-positive by routine diagnostics, whole-genome sequencing failed to confirm MSI in a subset. Most discordant patients did not benefit from immune checkpoint blockade. After detailed review, some discordances were explained by false-positive routine testing or by the presence of multiple primary tumors, while others represented dMMR tumors lacking a canonical MSI molecular phenotype. This work illustrates that incorrect attribution of the biomarker can lead directly to inappropriate immunotherapy exposure [29].

## 7. Plasma MSI Detection and the Tissue MSS/pMMR to ctDNA MSI-H Scenario

Liquid biopsy has expanded the ways in which MSI can be detected. In advanced GI cancers, the GOZILA substudy demonstrated that plasma-based MSI assessment using ctDNA had very high overall agreement and negative percent agreement versus tissue testing, but positive percent agreement depended strongly on ctDNA fraction, as defined by the maximum variant allelic fraction in this study. When ctDNA fraction was at least 1%, positive percent agreement reached 100% in that dataset, whereas lower-shedding tumors were more challenging. These results support plasma MSI as a clinically useful biomarker when tumor DNA fraction is adequate but also reinforce that plasma assays are not ideal arbiters in low-shedding settings [30,31].

Our clinical scenario, namely tissue MSS/pMMR CRC followed by ctDNA MSI-H, can therefore be interpreted in several ways. The first is straightforward sampling bias: the original tissue specimen may have missed an MSI-H region or may have had technical limitations. The second is that ctDNA may preferentially reflect a metastatic lesion or a later-emerging branch that was not represented in the diagnostic sample. The third is that the plasma assay detects a noncanonical unstable phenotype not visible on standard IHC. The fourth is that a different primary tumor, rather than the index CRC, contributes MSI-H ctDNA. CAP’s recent review explicitly flags this latter possibility, especially in patients with multiple primaries or hereditary predisposition [4,13].

Thus, a plasma MSI-H result after prior tissue MSS/pMMR testing should be taken seriously, but not at face value. The most defensible clinical response is confirmatory adjudication: re-review of the original pathology, orthogonal tissue testing if not already performed, biopsy of a current metastatic lesion if feasible, and broader genomic interrogation of the MMR pathway including MLH1 methylation and noncanonical repair genes.

Recent ctDNA analyses across solid tumors have demonstrated that biomarker clonality can influence therapeutic efficacy and may provide a quantitative measure of biologic relevance beyond simple biomarker presence or absence. These observations raise the possibility that future plasma MSI assays may need to assess not only whether MSI is present, but also the relative contribution of MSI-H clones to the overall ctDNA pool. Whether analogous measures of MSI clonality, unstable locus burden, or the proportion of ctDNA derived from MSI-H subclones influence response to immune checkpoint blockade remains unknown and warrants prospective study [26].

## 8. Immunotherapy Implications

The predictive value of MSI-H/dMMR for immunotherapy in canonical CRC is unequivocal. The original pembrolizumab studies in MMR-deficient tumors showed that MMR status, rather than tumor site, predicted response to PD-1 blockade. These observations led to tumor-agnostic approval and were later reinforced specifically in metastatic CRC by KEYNOTE-177, which demonstrated superior progression-free survival and better tolerability for first-line pembrolizumab versus chemotherapy in MSI-H/dMMR metastatic CRC [32]. More recently, nivolumab plus ipilimumab has shown strong activity and superior progression-free survival compared with nivolumab alone in the phase III CheckMate 8HW program [9,10,24].

The central issue for subclonal MSI is whether this benefit extends to tumors in which the unstable state is only partial, regional, or temporally emergent. Here, the most informative mechanistic work comes from Westcott and colleagues. They showed that mismatch repair deficiency alone is not sufficient to generate effective antitumor immunity; rather, intratumor heterogeneity can blunt T-cell responses, and the clonal, not merely total, neoantigen burden best correlates with response to immune checkpoint blockade in MMR-deficient GI cancers [33]. This has direct implications for the clinical meaning of a small MSI-H subclone. If the unstable branch constitutes only a minority of total disease burden, many neoantigens generated by that branch will be subclonal and absent from other lesions or compartments, limiting the breadth and durability of immune-mediated tumor control.

This principle likely explains why some discordant or noncanonical dMMR/MSI tumors do not behave like classic MSI-H CRC. The Drug Rediscovery Protocol (DRUP) is a pan-cancer prospective trial conducted in the Netherlands which evaluates off-label treatments for actionable genomic alterations in solid tumors. In the DRUP-related outcomes analysis by Verkerk and colleagues, most patients with routine dMMR/MSI positivity but no corresponding MSI phenotype by whole-genome sequencing did not benefit from ICI. These data suggest that immunotherapy sensitivity requires more than a nominally positive biomarker result [34].

The EBioMedicine pMMR/MSI-H CRC study showed that these tumors can have increased immune infiltration relative to pMMR/MSS CRC, implying that at least some apparently discordant tumors are biologically immunogenic. Likewise, recent clinicogenomic work showed that MSI-H CRCs with *B2M* or *JAK1/2* mutations should not be excluded categorically from PD-1 therapy, since benefit may still occur despite these immune-evasion alterations [35]. Thus, subclonal or atypical MSI should be viewed as a spectrum of uncertain but potentially meaningful checkpoint sensitivity rather than as uniformly responsive or uniformly resistant.

A practical inference is that the likelihood of immunotherapy benefit probably tracks with the clonal breadth of MMR deficiency. Tumors in which a dominant metastatic clone is clearly MSI-H/dMMR are plausible candidates for PD-1-based therapy even if the original primary specimen was reported as MSS/pMMR. By contrast, a low-level plasma MSI-H signal without corroborative tissue evidence, particularly in a patient with low ctDNA fraction or multiple possible primaries, should be interpreted cautiously [33]. However, these concepts remain largely biologic hypotheses rather than clinically validated biomarkers. At present, no quantitative measure of MSI burden or clonality has been shown to reliably predict response to immune checkpoint blockade, and clinical interpretation therefore remains dependent on the totality of molecular, pathologic, and clinical findings.

## 9. Proposed Clinical Approach to Suspected MSI/MMR Subclonality in CRC

When confronted with CRC initially labeled MSS/pMMR in tissue but later showing ctDNA MSI-H, the first step should be pathology adjudication rather than immediate therapeutic inference. Original slides and IHC should be re-reviewed with attention to internal controls, fixation quality, and abrupt regional loss patterns, particularly involving MSH6. If only one tissue block was tested originally, additional blocks should be examined when available, since historical and contemporary studies both show that heterogeneity can be missed by limited sampling. Orthogonal testing should then be performed using a second modality, such as PCR-based MSI or NGS-based MSI if IHC was the original assay, or vice versa.

If disease is metastatic and accessible, biopsy of a contemporary lesion is especially informative because it answers two clinically urgent questions at once: whether a current tumor site is truly MSI-H/dMMR and whether the evolving disease burden is dominated by an unstable clone. Broader genomic profiling should assess canonical MMR genes, *MLH1* promoter methylation, proofreading polymerase genes, and noncanonical repair genes such as *MSH3*. If the patient has a clinical context raising suspicion for multiple primaries, the possibility that ctDNA is reflecting a different malignancy should be explicitly considered.

**Figure A2:**
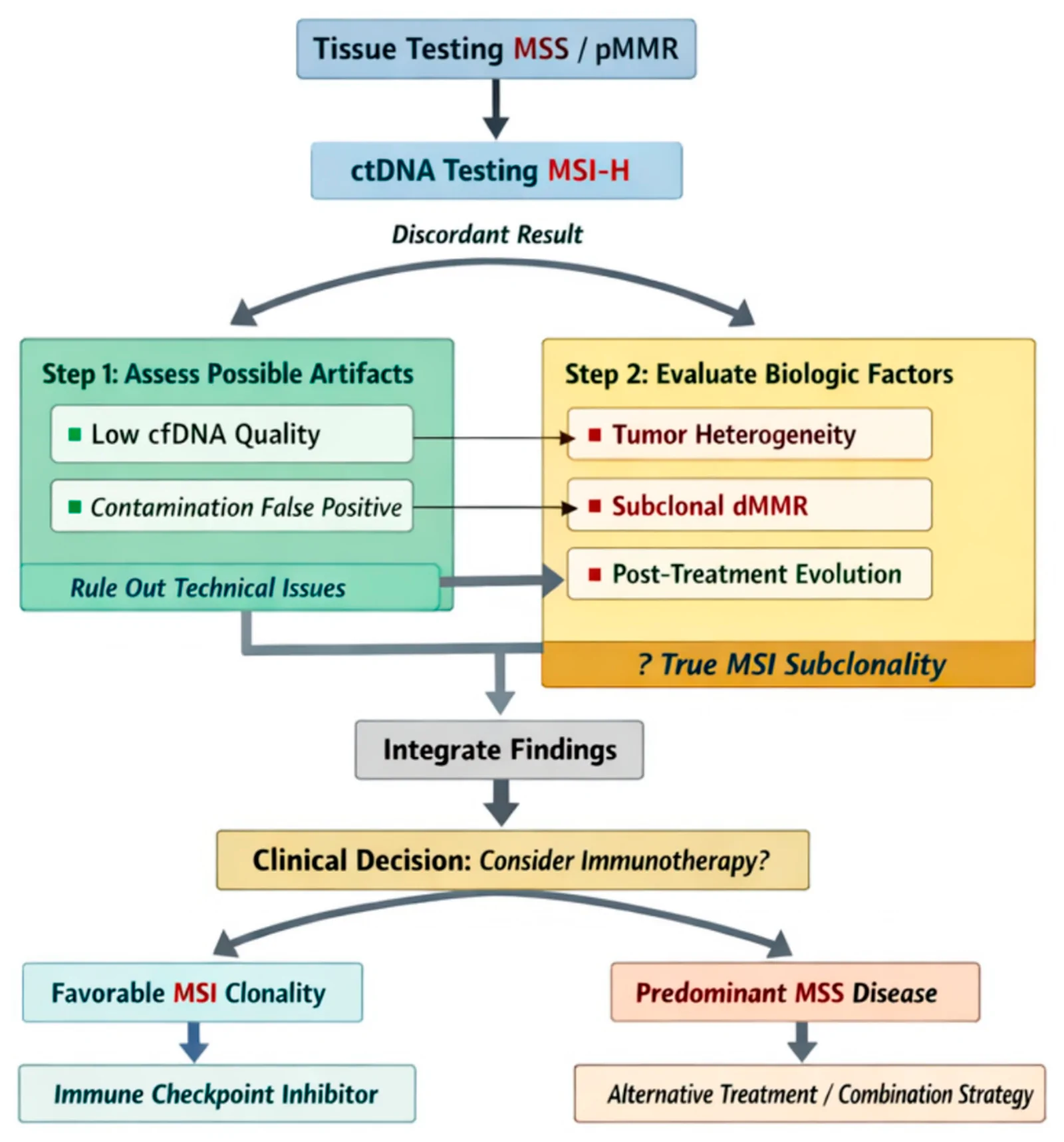
Diagnostic Algorithm for MSI Discordance.

For treatment decisions, definitive evidence of MSI-H/dMMR in a current metastatic lesion should carry substantial weight even if an older primary specimen was MSS/pMMR. CAP/ASCO guidance considers definitive dMMR by IHC or MSI-H by a validated molecular assay potentially sufficient for immune checkpoint inhibitor eligibility, but discordant results should first undergo expert review to exclude artifact, treatment effect, assay limitations, and a second primary tumor [13,17,28]. Isolated plasma MSI-H without corroborative evidence remains a gray zone. Treatment selection should be individualized and, whenever feasible, supported by repeat or region-directed tissue testing, serial ctDNA results, co-occurring genomic signatures of mismatch repair deficiency, and multidisciplinary review of the clinical context (Figure A2).

## 10. Future Directions

The field now needs to move beyond simple assay concordance studies toward a biologically richer understanding of unstable states. First, prospective multiregion sequencing of primary and metastatic CRC with matched plasma is needed to define how often tissue MSS/pMMR to plasma MSI-H truly reflects branched evolution rather than analytic discordance. Second, the quantitative threshold of clonal breadth required for checkpoint sensitivity remains unknown. Plasma-based assays may eventually help estimate not just whether MSI is present, but how much of the disease burden is contributing the unstable signal. Third, noncanonical and dynamic mutator states deserve more attention, particularly those involving *MSH3*, *MSH6*, polymerase genes, and epigenetic modulation. Fourth, immunotherapy trials in biomarker-atypical CRC should incorporate multiregion and longitudinal molecular sampling to determine whether partial or emergent MSI has predictive value when combined with TMB, neoantigen clonality, or immune infiltration metrics.

Finally, pathology reporting may need refinement. Binary labels such as intact versus lost or MSI-H versus MSS remain indispensable, but they are sometimes insufficient. Reporting frameworks that explicitly note abrupt regional loss, mosaic staining, equivocal or noncanonical patterns, and the recommendation for orthogonal adjudication would likely improve both hereditary cancer evaluation and immunotherapy triage.

## 11. Conclusions

MSI/MMR subclonality is a real but uncommon phenomenon in human cancers and in CRC specifically. The available evidence indicates that most apparent discordance between tissue and plasma, or between different assays, is explained by technical or sampling factors. Nevertheless, carefully studied cases prove that genuine regional or temporal heterogeneity can occur, that pMMR/MSI-H CRC represents a biologically meaningful subgroup, and that mutator states can evolve dynamically under selective pressure. The therapeutic consequences of these findings are substantial. Immunotherapy benefit in CRC appears to depend less on detecting MSI somewhere within the cancer than on whether MMR deficiency is clonally dominant enough to generate a broad, shared neoantigen repertoire and an immunologically engaged tumor microenvironment. Until more prospective data become available, the optimal response to suspected MSI/MMR subclonality is rigorous adjudication using multiregion pathology, orthogonal molecular testing, and contemporaneous sampling of metastatic disease when feasible. For molecular oncologists, the most important lesson is that MSI should no longer be regarded as an entirely binary attribute; in a minority of clinically challenging cases, it is better understood as an evolving, spatially structured, and therapeutically consequential feature of tumor evolution.

## Data Availability

The original contributions presented in this study are included in this article. Further inquiries can be directed to the corresponding author.

## Abbreviations

The following abbreviations are used in this manuscript:

CRC	Colorectal cancer
ctDNA	Circulating tumor DNA
dMMR	Deficient mismatch repair
IHC	Immunohistochemistry
MSI-H	Microsatellite instability-high
MSS	Microsatellite stable
pMMR	Mismatch repair proficient
